# Meta-analysis of genome-wide association study identifies *FBN2* as a novel locus associated with systemic lupus erythematosus in Thai population

**DOI:** 10.1186/s13075-020-02276-y

**Published:** 2020-08-08

**Authors:** Pattarin Tangtanatakul, Chisanu Thumarat, Nusara Satproedprai, Punna Kunhapan, Tassamonwan Chaiyasung, Siriwan Klinchanhom, Yong-Fei Wang, Wei Wei, Jeerapat Wongshinsri, Direkrit Chiewchengchol, Pongsawat Rodsaward, Pintip Ngamjanyaporn, Thanitta Suangtamai, Surakameth Mahasirimongkol, Prapaporn Pisitkun, Nattiya Hirankarn

**Affiliations:** 1 Department of Transfusion Sciences and Clinical Microbiology, Faculty of Allied Health Sciences, Chulalongkorn University, Bangkok, Thailand; 2 Section of Translational Medicine, Faculty of Medicine, Mahidol University, Ramathibodi Hospital, Bangkok, Thailand; 3 Department of Medical Sciences, Ministry of Public Health, Nonthaburi, Thailand; 4 Centre of Excellent in Immunology and Immune-Mediated Diseases, Department of Microbiology, Faculty of Medicine, Chulalongkorn University, 1873 Ratchadamri Road, Pathum wan, Bangkok, 10330 Thailand; 5 Department of Paediatrics and Adolescent Medicine, Faculty of Medicine, The University of Hong Kong, 21 Sassoon Road, Sandy Bay, Hong Kong; 6 Shenzhen Futian Hospital for Rheumatic Disease, Shenzhen, People’s Republic of China; 7 Lupus Research Institute, Affiliated Hospital of Jining Medical University, Jining, China; 8 Collaborative Innovation Centre for Birth Defect Research and Transformation of Shandong Province, Jining Medical University, Jining, China; 9 Department of Medicine, Nopparat Rajathanee Hospital, Bangkok, Thailand; 10 Division of Allergy, Immunology, and Rheumatology, Department of Medicine, Faculty of Medicine, Ramathibodi Hospital, Mahidol University, Bangkok, Thailand

**Keywords:** Genome-wide association study, Thai population, Systemic lupus erythematosus, Genetic susceptibility, Single nucleotide polymorphisms, Polygenic risk score

## Abstract

**Background:**

Differences in the expression of variants across ethnic groups in the systemic lupus erythematosus (SLE) patients have been well documented. However, the genetic architecture in the Thai population has not been thoroughly examined. In this study, we carried out genome-wide association study (GWAS) in the Thai population.

**Methods:**

Two GWAS cohorts were independently collected and genotyped: discovery dataset (487 SLE cases and 1606 healthy controls) and replication dataset (405 SLE cases and 1590 unrelated disease controls). Data were imputed to the density of the 1000 Genomes Project Phase 3. Association studies were performed based on different genetic models, and pathway enrichment analysis was further examined. In addition, the performance of disease risk estimation for individuals in Thai GWAS was assessed based on the polygenic risk score (PRS) model trained by other Asian populations.

**Results:**

Previous findings on SLE susceptible alleles were well replicated in the two GWAS. The SNPs on HLA class II (rs9270970, A>G, OR = 1.82, *p* value = 3.61E−26), *STAT4* (rs7582694, C>G, OR = 1.57, *p* value = 8.21E−16), *GTF2I* (rs73366469, A>G, OR = 1.73, *p* value = 2.42E−11), and *FAM167A-BLK* allele (rs13277113, A>G, OR = 0.68, *p* value = 1.58E−09) were significantly associated with SLE in Thai population. Meta-analysis of the two GWAS identified a novel locus at the *FBN2* that was specifically associated with SLE in the Thai population (rs74989671, A>G, OR = 1.54, *p* value = 1.61E−08). Functional analysis showed that rs74989671 resided in a peak of H3K36me3 derived from CD14+ monocytes and H3K4me1 from T lymphocytes. In addition, we showed that the PRS model trained from the Chinese population could be applied in individuals of Thai ancestry, with the area under the receiver-operator curve (AUC) achieving 0.76 for this predictor.

**Conclusions:**

We demonstrated the genetic architecture of SLE in the Thai population and identified a novel locus associated with SLE. Also, our study suggested a potential use of the PRS model from the Chinese population to estimate the disease risk for individuals of Thai ancestry.

## Background

The systemic lupus erythematosus (SLE) is a systemic autoimmune disease characterized by loss of tolerance to self-antigens, inappropriate immune activation, and inflammation [1]. The severity is various depending on affected organs [2]. Genetic susceptibility has been widely accepted as one of the critical factors driving disease development [2]. Recently, the genetic architecture of SLE has been examined worldwide [3]. Using GWAS, more than 90 loci have been found associated with SLE across at least four ethnic groups, including Han Chinese, European, North America, and Africa [4, 5]. The strongest signal was identified at the HLA class II allele, which replicated in all of the different populations [4]. These findings indicate critical biological mechanisms underlying the disease, which will be the candidate in further functional studies [6].

However, heterogeneity of disease between different ethnicities drives a question of whether genetic background in different ancestries could affect the clinical manifestations. It is known that Asian SLE patients have higher disease severity compared to Europeans [2]. However, only a few studies on SLE associations that were based on candidate genes were performed in the Thai population [7–11]. In this study, we conducted GWAS using the SLE samples collected from two tertiary referral hospitals in Thailand. We aim to replicate known SLE-associated variants in the Thai population and identify novel SNPs associated with SLE.

## Methods

### Sample collection and preparation

We calculated the power of our study by using an online tool called Genetic association study (GAS) Power Calculator [12]. With 800 cases and 1600 controls at 5E−08 significant level, we obtained 0.934 expected power for the study. The EDTA blood samples from SLE patients (*n* = 487) were collected at King Chulalongkorn Memorial Hospital as cases for the discovery phase. All procedures were approved by the ethical committee from the Faculty of Medicine, Chulalongkorn University (COA no. 923/2017). For the replication cohort, the samples (*n* = 405) were collected from the Rheumatology clinic, Ramathibodi Hospital, with ethical approval from the Faculty of Medicine, Mahidol University (MURA no. 2015/731, EC no. 590223, Protocol-ID 12-58-12). All patients were carefully recruited regarding the criteria from the American College of Rheumatology (ACR) [13]. Patients’ demographic data from both datasets have been summarized in Table 1. For healthy controls (*n* = 1606) and unrelated disease controls including breast cancer, periodontitis, tuberculosis, drug-induced liver injury, epileptic encephalopathy, dengue hemorrhagic fever, thalassemia, and cardiomyopathy (*n* = 1590), data were provided from the Department of Medical Science, Ministry of Public Health, Thailand.

**Table 1:** SLE patients’ characteristics of both observatory and replication datasets

Patients’ characteristics	Clinical cases			
	Observatory cohort ***n*** = 455^**a**^		Replication cohort ***n*** = 371^**a**^	
	***n***	(%)	***n***	(%)
**Age of onset (mean ± SD)**	30.38	± 13.68	30.39	± 11.43
**Sex**				
Female	425	(93.41%)^b^	337	(90.84%)^c^
Male	26	(5.71%)^b^	27	(7.28%)^c^
**Clinical aspects**				
Hemologic disorders	243	(53.41%)^b^	136	(36.66%)^c^
Neurological disorders	62	(13.63%)^b^	33	(8.89%)^c^
Ulcer	115	(25.27%)^b^	52	(14.02%)^c^
Discoid rash	161	(35.38%)^b^	49	(13.21%)^c^
Malar rash	142	(31.21%)^b^	82	(22%)^c^
Arthritis	133	(29.23%)^b^	148	(39.89%)^c^
Renal disorders	284	(62.42%)^b^	149	(40.16%)^c^
ANA	350	(76.92%)^b^	214	(57.68%)^c^

^a^The sample number after quality control processes

^b^The percentages of unknown clinical data (n/a) in the observatory dataset are listed here. Sex = 0.88%, hematologic disorder = 1.76%, neurological disorder = 2.20%, ulcer = 4.18%, discoid rash = 3.96%, malar rash = 5.71%, arthritis = 4.18%, renal disorders = 1.76%, and ANA = 9.89%

^c^The percentages of unknown clinical data (n/a) in the replication dataset are listed here. Sex = 0.00%, hematologic disorder = 36.93%, neurological disorder = 37.2%, ulcer = 37.4%, discoid rash = 37.2%, malar rash = 37.47%, arthritis = 37.2%, renal disorders = 37.74%, and ANA = 36.93%

### DNA extraction

Buffy coats were extracted using the QIAGEN® EZ1® DNA blood kit (QIAGEN GmbH, Hilden, Germany). We used 200 μl of a buffy coat as recommended by the manufacturer’s instruction. Buffy coat samples were transferred into tube or sample cartridge for EZ1 Advanced XL (QIAGEN GmbH, Hilden, Germany) and extracted using EZ1® Advanced XL DNA Buffy coat protocol. From this protocol, DNA was eluted at 200 μl. DNA was diluted and quantitated using Qubit™ dsDNA BR Assay Kit according to the manufacturing protocol (Invitrogen, Thermo Fisher Scientific, MA, USA).

### Genotyping and quality control

**Fig. 1:**
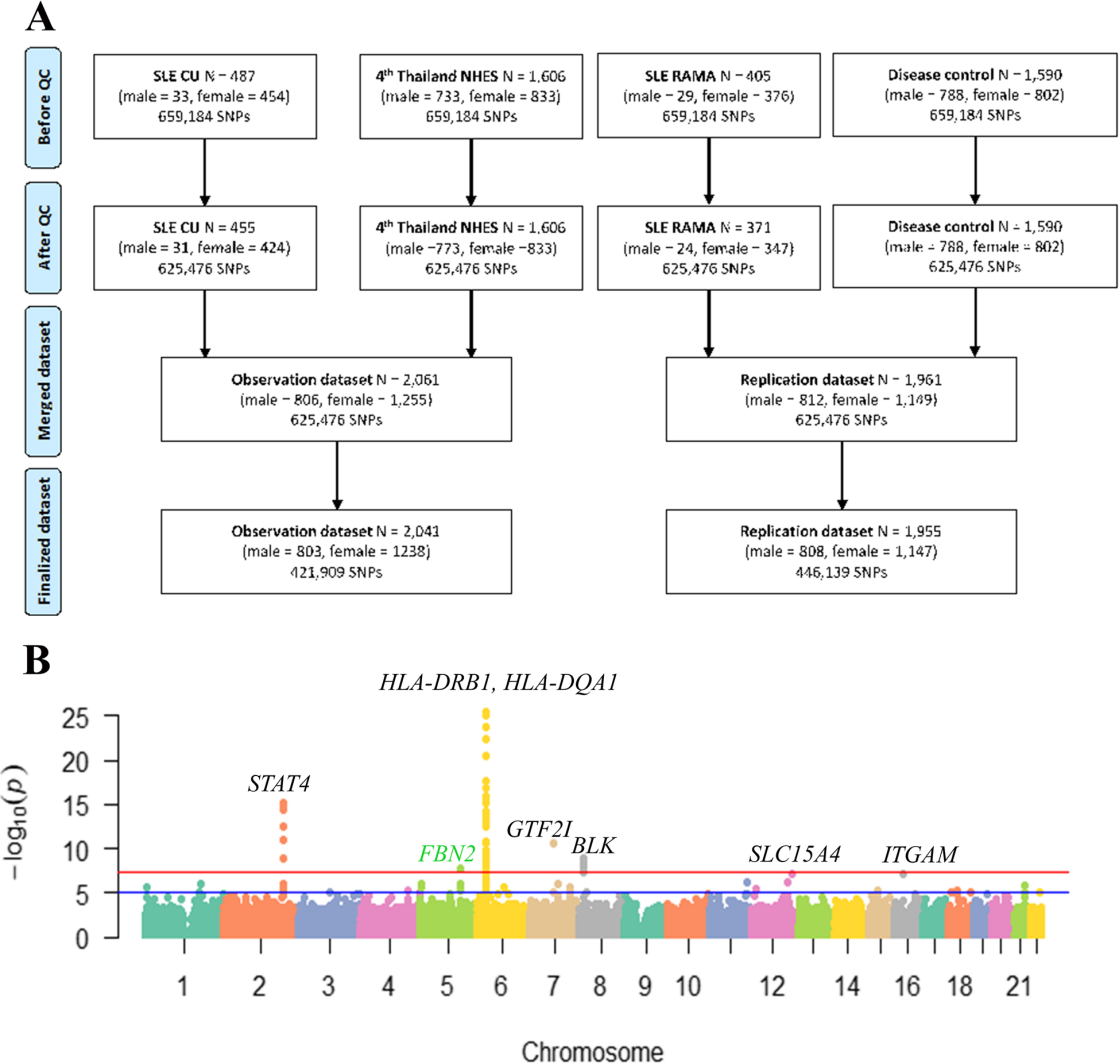
Quality control and dataset preparation flow diagram of both discovery and validation datasets. The flow diagram was modified from the PRISMA flow diagram [15] (**a**). Manhattan plot on the meta-analysis result of the two SLE GWAS datasets in the Thai population using R-Bioconductor package qqman (**b**)

Genotyping was performed using Infinium Asian Screening Array-24 v1.0 BeadChip with 659,184 SNPs (Illumina, San Diego, CA, USA) at the Department of Medical Sciences (DMSC, Ministry of Public Health, Thailand) based on the protocol recommended by the manufacturer. The Genome Studio data analysis software v2011.1 (Illumina, San Diego, CA, USA) was used for calling genotypes. Samples and SNP markers were tested for quality control (QC) using PLINK genomic analysis software (v1.90b5.4) [14]. Individuals with autosomal genotype call rate ≤ 0.98, gender inconsistency based on heterozygosity rate of X chromosome (maleTh = 0.8, femaleTh = 0.2), and genome-wide estimates of identity-by-descent (pihat) ≥ 0.185 (3rd generation) were excluded from analysis. SNPs with more than 5% missing genotyping rate or significant deviation of Hardy-Weinberg equilibrium (*p* value ≤ 1 × 10^−8^) were also removed. After quality control (QC), we obtained a dataset of 2041 individuals with 421,909 variants for the discovery phase and 1955 individuals with 446,139 variants for replication. The flow diagram of the analysis process is shown in Fig. 1a.

### GWAS data imputation

Pre-phasing was performed using SHAPEIT [16]. After that, genotype data for individuals was imputed to the density of the 1000 Genomes Project Phase 3 reference using IMPUTE2 [17]. After all the QC processing, 6,657,806 were left for further analysis. The processed data were publicly available at http://2anp.2.vu/GWAS_SLE_Thailand.

### Association study, meta-analysis, and statistical analysis

The association studies were conducted by using SNPTEST [18], and the factored spectrally transformed linear mixed models (FaST-LMM v.0.2.32) program [19]. The results from FaST-LMM were analyzed and visualized by RStudio to obtain genomic inflation factor (*λ*), quantile-quantile plot, and Manhattan plot [20]. The SNPs with *p* value ≤ 1 × 10^−5^ were plotted to obtain the regional plot by using LocusZoom [21]. Haplotype block and linkage disequilibrium structure were analyzed by Haploview software version 4.2 [22]. The characterized SLE susceptible loci were downloaded from a previous study [23] and GWAS catalogue (the NHGRI-EBI catalogue of published genome-wide association studies). Meta-analysis was studied based on the inverse variant strategy in the METAL program [24]. The genetic inheritance pattern was calculated from the frequency of different genotyping on risk alleles using R-Bioconductor. Simultaneously, functional annotation was predicted by using SNPnexus, which applied data from the Reactome database [25]. The histone markers and transcription factor binding sites were predicted from an online tool called HaploReg V4.1 [26].

### Polygenic risk score calculation

Lassosum [27] was used to calculate PRS for individuals. The summary statistics for SLE association in East Asians [28], involving 2618 cases and 7446 controls with Chinese ancestry, were used to train the model. The area under the ROC curve (AUC) was calculated using R package pROC [29].

## Results

### Known SLE associations found in the Thai population

In the discovery dataset, the association studies were initially performed using healthy controls (*n* = 1606) and SLE patients (*n* = 487) collected from King Chulalongkorn Memorial Hospital. Regarding the result, we found that variants at the HLA class II regions were strongly associated with SLE (*p* value < 5E−08). Similarly, GWAS from 405 SLE cases and 1590 non-immune-mediated disease controls found variants at the HLA class II regions reached the genome-wide significant threshold (*p* value < 5E−08). Our findings were consistent with previous reports in other ethnic groups [30]. Inflation factors from both datasets were calculated as reported in Supplementary figure 1.

Subsequently, a meta-analysis of the two Thai GWAS was carried out, and we systematically examined associations across the 90 known SLE-associated loci, which were collected from the GWAS catalogue (https://www.ebi.ac.uk/gwas/) and previous review articles [23]. Of these loci, the *HLA-DQA1*, *HLA-DRB1*, *STAT4*, *FAM167A-BLK*, and *GTF2I* loci have reached the genome-wide significant threshold (*p* value < 5E−08; Fig. 1b, Table 2) in Thai population, and the variants at the *PROS1C1*, *NOTCH4*, *HCP5*, *C6orf10*, *TAP2*, *TNFSF4*, *RasGRP3*, *TERT*, *TNPO3-IRF5*, *CXCR5*, *GPR19*, *SLC15A4*, and *ITGAM* loci showed suggestive evidence of associations with SLE (*p* value < 5E−05, Supplementary Table 1). These loci have been found in several ancestries, including Han Chinese, Korean, North American, European, African, and Hispanic populations [31, 32].

**Table 2:** List of significant variants at individual locus from the meta-analysis

HAP^**a**^	dbSNP^**b**^	CHR^**c**^	BP^**d**^	RA^**e**^	MAF affected	MAF unaffected	Locus	Locus upstream	Locus downstream	Discovery dataset		Replication dataset		Meta-analysis		***p***_**het**_^**f**^
										OR (95% CI)	***p***	OR (95% CI)	***p***	OR	***p***	
q32.3	rs7574865	2	191,099,907	A	0.47	0.36	STAT4			1.54 (1.33–1.79)	1.45E−08	1.61 (1.37–1.89)	7.45E−09	1.57	8.218E−16	0.69
q23.3	rs74989671	5	128,398,268	G	0.16	0.11	FBN2			1.52 (1.24–1.86)	4.31E−05	1.58 (1.26–1.98)	7.61E−05	1.54	1.611E−08	0.81
p21.32	rs9270970	6	32,605,797	G	0.42	0.30		HLA-DRB1	HLA-DQA1	2.02 (1.73–2.35)	8.71E−20	1.63 (1.39–1.93)	4.15E−09	1.83	3.617E−26	0.07
q11.23	rs73366469	7	74,619,286	G	0.14	0.09		RP5-1186P10.2	GTF2I	1.8 (1.45–2.24)	1.09E−07	1.65 (1.3–2.1)	2.84E−05	1.73	2.42E−11	0.61
p23.1	rs13277113	8	11,491,677	G	0.26	0.32		FAM167A	BLK	0.64 (0.54–0.76)	2.16E−07	0.74 (0.61–0.88)	8.76E−04	0.68	1.58E−09	0.27
q24.33	rs1385374	12	128,816,149	A	0.20	0.15	SLC15A4			1.54 (1.28–1.85)	5.76E−06	1.37 (1.12–1.69)	2.36E−03	1.46	7.62E−08	0.43
p11.2	rs1143679	16	31,265,490	A	0.07	0.03	ITGAM			1.67 (1.21–2.28)	1.39E−03	2.27 (1.6–3.23)	2.55E−06	1.91	5.81E-08	0.2

^a^Haplotype

^b^dbSNP from single nucleotide polymorphisms database (NCBI)

^c^Chromosome

^d^Position

^e^Risk alleles

^f^*p* value of heterogeneity

We noticed that some of the previously characterized nonsynonymous polymorphisms also showed certain evidence of association (*p* value < 0.05) in Thai population, such as rs11235604 (*ATG16L2*, R58W), rs13306575 (*NCF2*, R395W), rs1990760 (*IFIH1*, A946T), rs3734266 (*UHRF1BP1*, Q454L), rs2841280 (*PLD4*, E27Q), and rs2230926 (*TNFAIP3*, F127S). Details of these associations are summarized in Table 3. All significant variants were calculated for Hardy-Weinberg equilibrium, as reported in Supplementary Table 2.

**Table 3:** List of known SLE susceptible SNPs in Thai SLE patients

dbSNP^a^	CHR^b^	BP^c^	RA^d^	Locus	Annotation	MAF affected	MAF unaffected	OR	SE	*p*
rs35426045	1	161,649,724	A	FCGR2B	Intergenic	0.80	0.75	1.38	0.09	1.83E−04
rs1234315	1	173,209,324	A	TNFSF4	Intergenic	0.53	0.46	1.27	0.07	1.02E−06
rs2205960	1	173,191,475	T	TNFSF4	Intergenic	0.27	0.22	1.26	0.08	2.37E−03
rs34889541	1	198,594,769	A	ATP6V1G3,	Intergenic	0.10	0.13	0.75	0.11	7.66E−03
rs1418190	1	173,361,979	T	LOC100506023	ncRNA_intronic	0.59	0.56	1.18	0.07	1.55E−02
rs13306575	1	183,563,302	A	NCF2	Nonsynonymous	0.11	0.08	1.48	0.09	1.73E−02
rs13385731	2	33,701,890	C	RASGRP3	Intronic	0.13	0.17	0.70	0.09	1.71E−05
rs6705628	2	74,208,362	T	DGUOK-AS1	ncRNA_exonic	0.11	0.13	0.79	0.10	1.83E−02
rs1990760	2	163,124,051	T	IFIH1	Missense	0.23	0.21	1.17	0.08	4.93E−02
rs10936599	3	169,492,101	T	MYNN	Synonymous SNV	0.52	0.56	0.84	0.07	6.95E−03
rs564799	3	159,728,987	T	IL12A	ncRNA_intronic	0.12	0.14	0.80	0.10	1.97E−02
rs10028805	4	102,737,250	A	BANK1	Intronic	0.45	0.49	0.87	0.07	4.08E−02
rs7726159	5	1,282,319	A	TERT	Intron	0.43	0.40	1.25	0.07	5.00E−05
rs2736100	5	1,286,401	C	TERT	Intron	0.51	0.43	1.25	0.07	4.67E−05
rs10036748	5	150,458,146	T	TNIP1	Intronic	0.66	0.61	1.16	0.07	3.04E−02
rs2431697	5	159,879,978	C	PTTG1; MIR146A	Intergenic	0.07	0.09	0.77	0.13	3.36E−02
rs548234	6	106,568,034	T	PRDM1; ATG5	Intergenic	0.67	0.72	0.81	0.07	2.21E−03
rs2230926	6	138,196,066	G	TNFAIP3	Missense	0.04	0.03	1.49	0.18	2.92E−02
rs3734266	6	34,823,187	C	UHRF1BP1	Intronic	0.21	0.19	1.18	0.08	4.68E−02
rs4728142	7	128,573,967	A	KCP; IRF5	Intergenic	0.19	0.13	1.61	0.09	1.34E−07
rs729302	7	128,568,960	C	KCP; IRF5	Intergenic	0.25	0.30	0.77	0.07	3.32E−04
rs12531711	7	128,617,466	G	IRF5; TNPO3	Intron	0.03	0.01	2.03	0.25	4.27E−03
rs4917014	7	50,305,863	G	C7orf72; IKZF1	Intergenic	0.15	0.17	0.81	0.09	1.84E−02
rs7097397	10	50,025,396	A	WDFY4	Missense	0.59	0.64	0.78	0.07	3.84E−04
rs4948496	10	63,805,617	C	ARID5B	Intronic	0.66	0.62	1.17	0.07	2.19E−02
rs1128334	11	128,328,959	T	ETS1	UTR3	0.35	0.28	1.36	0.07	1.50E−05
rs2732552	11	35,084,592	C	PDHX	Intergenic	0.78	0.75	1.18	0.08	3.04E−02
rs11235604	11	72,533,536	T	ATG16L2	Missense	0.04	0.05	0.70	0.17	3.93E−02
rs1385374	12	129,300,694	T	SLC15A4	Intronic	0.21	0.15	1.46	0.09	7.62E−08
rs10845606	12	12,834,894	A	GPR19	Intronic	0.32	0.37	0.75	0.07	3.19E−06
rs2841280	14	105,393,556	C	PLD4	Nonsynonymous	0.52	0.45	1.91	0.07	5.81E−08
rs1143679	16	31,276,811	A	ITGAM	Missense	0.07	0.04	1.71	0.14	6.18E−08
rs11860650	16	31,315,385	A	ITGAM	Intronic	0.09	0.07	1.74	0.10	4.64E−03
rs1170426	16	68,603,798	T	ZFP90	Intronic	0.69	0.73	0.82	0.07	5.91E−03
rs7444	22	21,976,934	C	UBE2L3	UTR3	0.64	0.60	1.17	0.07	1.81E−02
rs463426	22	21,809,185	C	HIC2; TMEM191C	Intergenic	0.38	0.40	0.85	0.08	4.50E−02

^a^dbSNP from single nucleotides polymorphisms database (NCBI)

^b^Chromosome

^c^Position

^d^Risk alleles

### Identification of novel loci associated with SLE

**Fig. 2:**
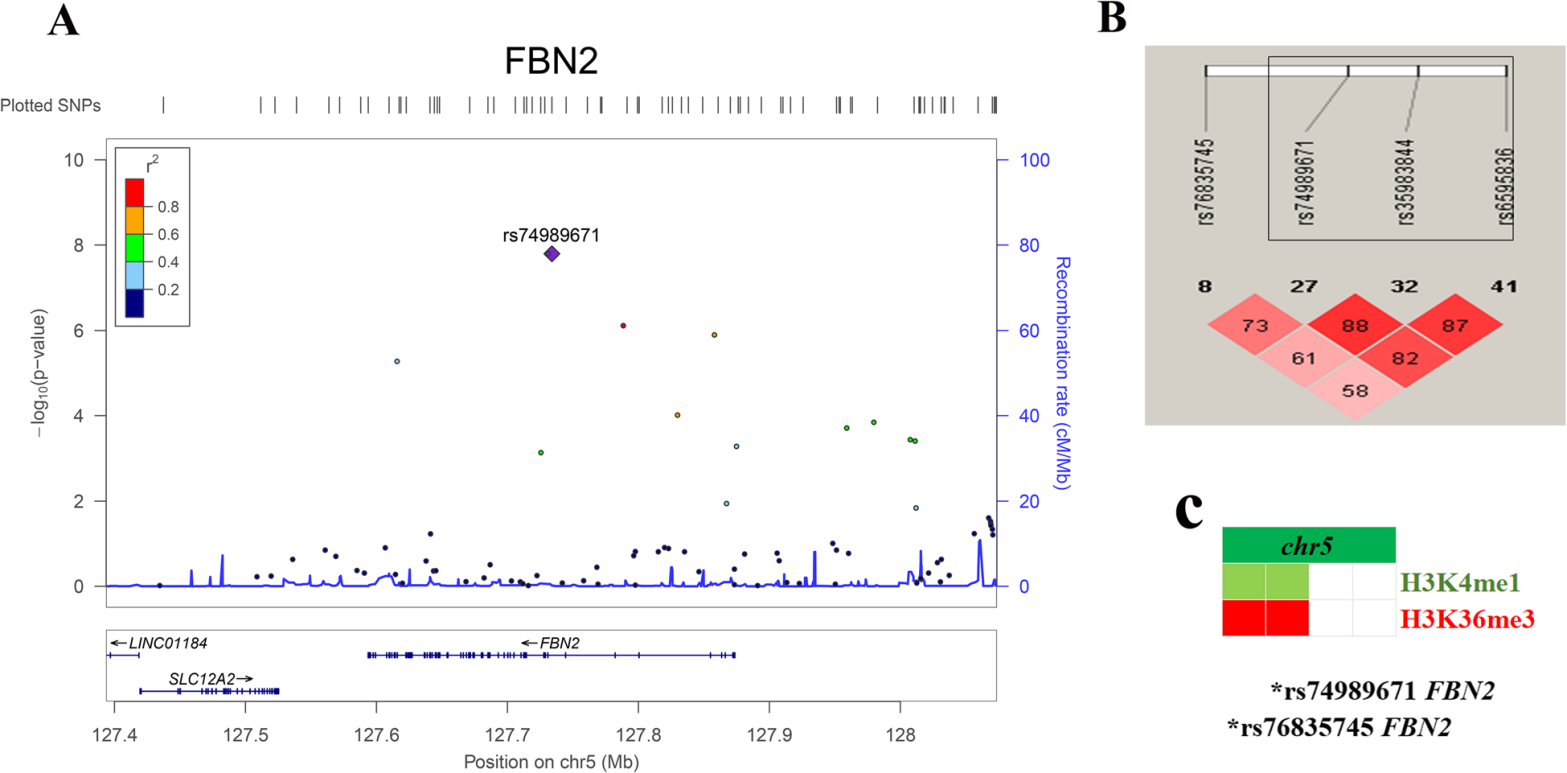
Regional plot of novel SLE susceptible variants on *FBN2* locus with their relative variants around *FBN2* locus (**a**). Haplotype block of significant variants on *FBN2* locus with their correlation to show linkage disequilibrium between SNPs (**b**). The picture illustrated histone markers overlapped with *FBN2* SNP site (**c**)

Excluding the variants at the known SLE-associated loci, we discovered a novel variant on *FNB2* (rs74989671, OR = 1.54, *p* value = 1.61E−08) specifically associated with SLE in Thai population (Figs. 1b and 2a, Table 2) when comparing the association in Europeans (OR = 0.998, *p* value = 0.979) and in Chinese populations (OR = 0.982, *p* value = 0.692) [28]. Further analyses based on different genetic inheritance models suggested that the disease risk was associated with the copy number of risk alleles that the individuals carried (additive model) (Table 4). Three SNPs on *FBN2* loci (rs74989671, rs35983844, rs6595836) showed linkage disequilibrium (LD *r*^2^ = 0.82) (Fig. 2b, Supplementary Table 1). Of these variants, rs74989671 was found to locate within the peak of H3K36me3 derived from CD14-positive monocytes and H3K4me1 (associated with active enhancers) derived from the primary T cells (Fig. 2c).

**Table 4:** Analyses based on different inheritance models on the *FBN2* locus

Locus SNPs	Model	Genotypes or alleles	SLE *n*	Control *n*	OR	95% CI	*p*
FBN2	Codominant	GG	21	26	1.75	0.93–3.27	7.96E−02
rs74989671	Dominant	AG	235	334	1.53	1.25–1.86	2.38E−05
		AA	562	1219	**ref**	**ref**	**ref**
		AG+GG	256	360	1.54	1.27–1.87	8.83E−06
		AA	562	1219	**ref**	**ref**	**ref**
	Recessive	GG	21	26	1.57	0.84–2.93	0.161
		AG + AA	797	1553	**ref**	**ref**	**ref**
	Allelic	A	277	386	**ref**	**ref**	**ref**
		G	1359	2772	1.38	1.17–1.64	1.31E−04
FBN2	Codominant	GG	655	1366	0.72	0.23–2.47	5.80E−01
rs76835745	Dominant	AG	162	212	1.15	0.36–4	1.00
		AA	6	9	**ref**	**ref**	**ref**
		GG+GA	817	1578	0.78	0.25–2.66	0.60
		AA	6	9	**ref**	**ref**	**ref**
	Recessive	GG	655	1366	0.63	0.5–0.79	5.43E−05
		AA+GA	168	221	**ref**	**ref**	**ref**
	Allelic	A	174	230	**ref**	**ref**	**ref**
		G	1472	2944	12.34	10.6–14.4	2.20E−16

In addition, we found variants at the *ATP6V1B1*, *MIR4472-2*, *MYO5C*, *ADCY5*, and *DGKG*, showing suggestive evidence of associations with SLE in Thai population (*p* value < 5E−05) (Supplementary Figure 2, Supplementary Table 1). Though these polymorphisms are likely to associate with Thai SLE patients, an independent GWAS dataset of SLE patients with Thai background is needed for further validation.

### In silico functional annotation of SLE-associated variants in Thai population

**Fig. 3:**
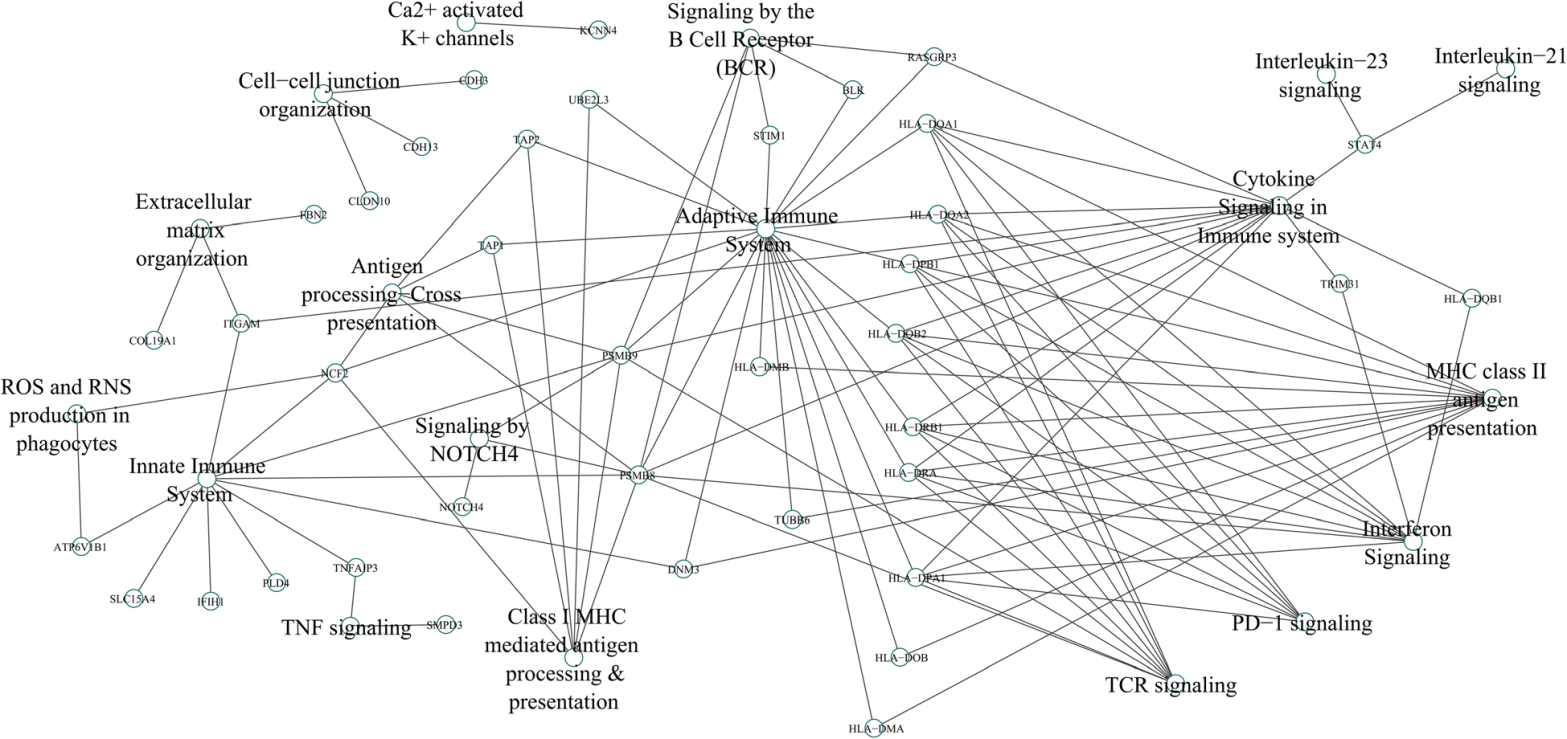
Diagram plot showed enrichment pathway from functional annotation analysis of significant variants (*p* value < 5E−05) using SNPnexus

To understand the biological meaning underlying the SLE-associated loci in the Thai population, we performed the pathway analysis using the SNPnexus program [25]. Variants with *p* value < 5E−05 were involved in this study. Notably, we found that 50% of all variants were located within the coding region, by which 10% is nonsynonymous polymorphisms. Pathway analysis results revealed that SLE-associated variants were highly enriched in the regulation of interferon signaling, PD-1 signaling, MHC-class II antigen presentation, TCR/BCR signaling, cytokine signaling, TNF signaling, NOTCH4 signaling, calcium-activated potassium channels, and cell-cell junction organization pathways. Furthermore, we found that extracellular matrix organization was significant in our results (Fig. 3). It indicated that Thai SLE patients might have a higher risk of fibrosis-associated inflammation.

### Polygenic risk score prediction for the individuals

**Fig. 4:**
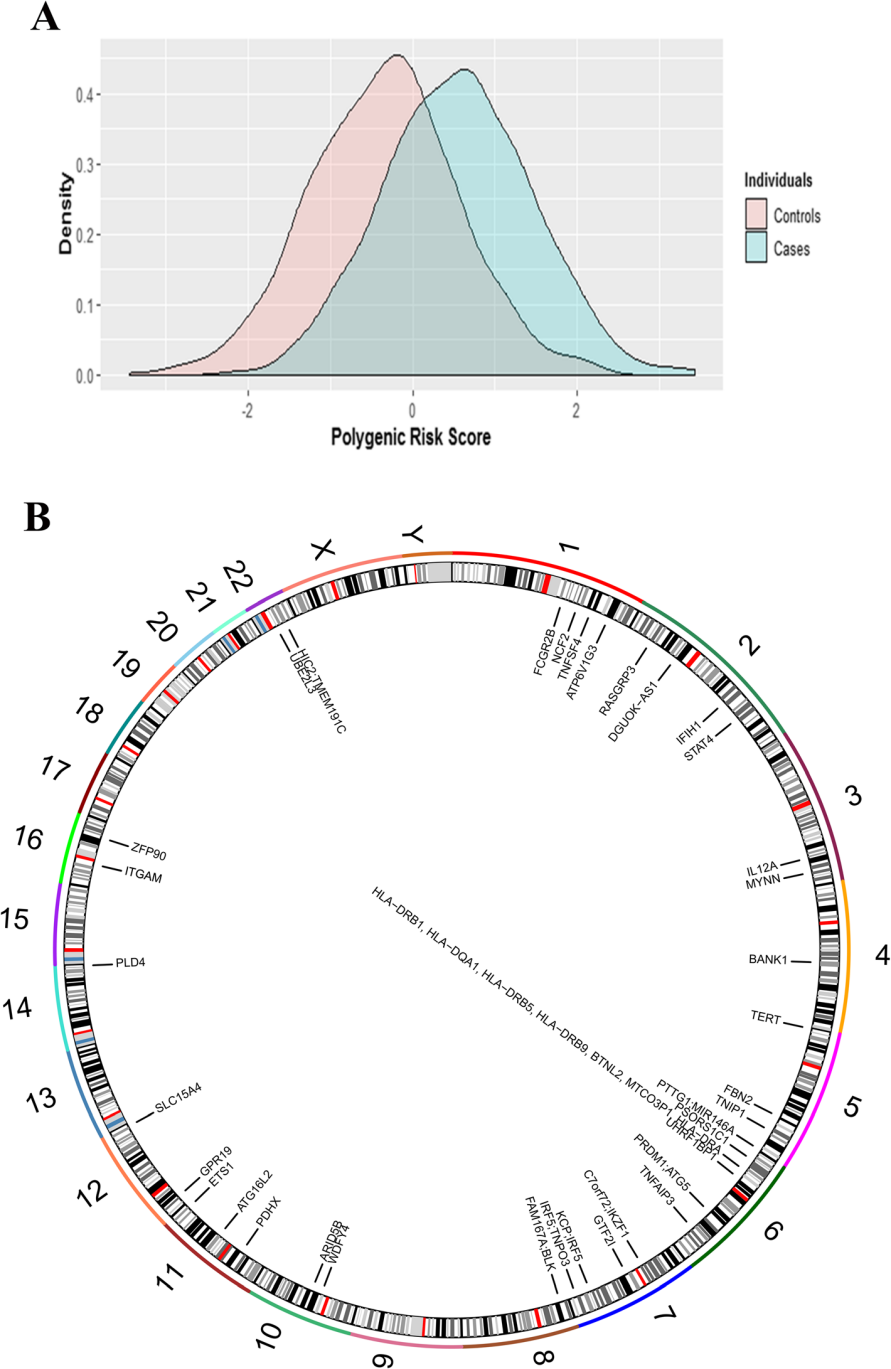
The graph shows the polygenic risk score calculation and the mean difference between SLE and healthy controls (**a**). The circular plot showed loci which identified in this study at individual chromosomes using package Rcircos [33] (**b**)

To apply the GWAS result to predict the Thai SLE outcome, we also tested the hypothesis of whether the PRS models trained by individuals with Chinese ancestry could be applied for Thai SLE patients. We calculated PRS for individuals in the Thai GWAS, based on the training data from the Chinese population (2618 cases and 7446 controls) [28]. Significantly, the PRS for SLE cases were higher than controls (mean difference = 0.89; *p* value = 2.2E−16; Fig. 4a), and the area under the receiver-operator curve (AUC) achieved 0.76 for this predictor. This analysis indicated the potential application for the PRS in the Thai population, based on the results from other Asian populations. Regarding the analysis, this might be a clue for predicting an outcome of SLE clinical characteristics in Thai SLE patients, and it is a good source for further genetic analysis to identify actual SLE pathogenesis in the different ancestry.

### Discussion

The present study is the first largest GWAS cohort conducted among Thai SLE patients. The highest significant association in the region of HLA class II was consistent with previous reports from other ethnic groups [30]. Since there are vast differences of HLA class II allele frequency among populations and sophisticated genetic structure, the study of the specific HLA class II haplotype is needed. Currently, there were two publications reported about specific HLA haplotypes in Thai SLE patients. First, the fine genetic mapping of the HLA allele from SLE patients in the northern part of Thailand has identified the association of HLA-DRB5*01:01 and HLA-DRB1*16:02 [34]. Secondly, HLA haplotype analysis found HLA-DRB1*15:02 and HLA-DQ*05:01 associated with Thai SLE patients [35]. Further study on the HLA class II allele using whole-genome sequencing or exome sequencing might be helpful to specify the impact of the HLA class II allele on Thai SLE patients.

Apart from the HLA class II alleles, our study found variants in *STAT4*, *GTF2I*, and *BLK* regions*.* For *BLK* locus, this gene encoded for Src-tyrosine kinase, which is an important signaling molecule under B cell development [36]. This gene showed protein-protein interaction with BANK1 (B cell-specific cytoplasmic protein involved in B cell receptor signaling) and might plausibly involve in dysregulation of the B cell receptor, which is a common feature found in SLE patients [37]. For *STAT4* and *GTF2I* alleles, these genes are encoded for the transcription factors that mediate many immune-related genes and inflammatory cytokine transcription machinery. Both *BLK* and *STAT4* loci have been reported as SLE susceptible alleles in Thai SLE patients recently [7], whereas *GTF2I* locus has firstly identified in our study. Interestingly, the variants on *STAT4* and *GTF2I* loci were correlated with lupus nephritis (LN) in the various SLE ancestries [32]. The *GTF2I* allele was likely to be specific in Asian background, mainly Han Chinese [38].

Our analysis found several LN-susceptible loci such as *IRF5* [39, 40], *ITGAM* [9, 41], *IKZF1* [42], and *TNFSF4* [43]. While IKZF1 is a co-transcription factor with STAT-4 mediated Th1 lymphocyte differentiation and interferon pathways [44], the *TNFSF4* locus, also called OX40L, encoded for the TNF superfamily ligand, which actively stimulates CD4+ T cell activation [43]. Study in the Finnish and Swedish SLE patients found the correlation of *ITGAM* with cutaneous discoid lupus erythematosus (DLE) and LN as well as Ro/SSA auto-antibody positive [45]. Not only LN, but we also found several loci that have been verified in the specific sub-phenotype of SLE patients. For example, our result found a variant on *ETS1*, which previously showed association with juvenile SLE, as well as a variant on *RasGRP3*, which was involved in malar rash or discoid rash [42]. The recent SLE susceptible loci identified in the cardiac manifestation of neonatal lupus, *NOTCH4*, was found in our results [46].

Note that we found some of the known SNPs which are nonsynonymous variants such as *NCF2* [47], *IFIH1* [48], *TNFAIP3* [49], *UHRF1BP1* [50], *ATG16L2* [51], and *PLD4* [52]*.* A few pieces of evidence have revealed the impact of those variants on various pathways including neutrophil extracellular traps (NETs) formation [53], sensor molecule to detect viral genome inside cells [54], a negative regulator for NF-kB transcription factors [55], and a negative regulator of cell growth [56]. These pathways resembled with our functional enrichment pathways analysis. Interestingly, our results found extracellular matrix organization (ECM) pathways associated with Thai SLE patients. Previously, single-cell transcriptome analysis in non-responder LN patients highlighted the upregulated genes in the ECM pathway correlated with treatment failure [57]. The ECM reflected the active fibrotic process, which was a marker of poor prognosis LN [58]. Remarkably, the prevalence of severe LN was high in the South East Asian ethnic included Thai [59]. As regards our SLE patients’ demographic data, we found that the frequency of clinical phenotype was roughly similar to other ethnic [60]. The LN has the highest abundance found among Thai SLE patients. Thus, our results supported that genetic background was a pivotal factor driving a severe LN among Thai SLE patients. Taken together, these pieces of evidence could justify the link between genetic variants and clinical involvement in Thai SLE patients.

The study of known SNPs showed most of the polymorphisms resembled with previous reports in Thais, such as *ARID5B*, *TNFSF4*, *BANK1*, *TNFAIP3*, *CXCR5 SLC15A*, *ITGAM*, *WDFY4*, *ETS1*, and *BLK* [7–11]. It confirmed that our analysis processes were reliable. Noticeably, the allele frequency of *ITGAM* was higher among Thai SLE when compared to Chinese Hong Kong [9], but has no association with Japanese and Korean background [61]. Thus, this implies the specificity of these variants to the Thai SLE patients. Although we did not recognize polymorphisms on chromosome 11q23.3 (rs11603023 on *PHLDB1* and rs638893 on *DDX6*), which has been identified in the Thais’ SLE, our meta-analysis enhanced signal from rs10845606 on *GPR19* allele which does not correlate with Thai SLE patients previously [8].

It is noteworthy that meta-analysis in the Thai population discovered novel SLE susceptible variants on *FBN2*. The *FBN2* allele is located on a chromosome 5 encoded protein called fibrillin-2 [62]. Fibrillins-2 is one of the glycoprotein components incorporated extracellularly on microfibrils and is essential in bone, muscle, and extracellular matrix formation [63]. It is well known that mutation of *FBN2* leads to dominant heritable connective tissue disorders [64]. Importantly, a recent review article has gained insight on fibrillin-2 as a critical mediator that binds to transforming growth factor-beta (TGF-β) during extracellular matrix formation [65]. The TLR9/TGF-β1/PDGF-β pathway was excessively activated in peripheral mononuclear cells isolated from LN patients [66]. Besides, the upregulation of *FBN2* correlated with fibrosis prevalence in the spontaneous LN developed mouse model (SWR X NZB1 F1) [67]. Although the function of *FBN2* in SLE is unclear, collective evidence led us to hypothesize that this variant might drive either fibrosis-associated inflammation or inflammatory induction during disease pathogenesis. Due to whole-genome sequencing data in the Thai population is lacking, further study using *FBN2* target sequencing, whole-genome sequencing, and variant functional characterization in a large cohort is needed. This knowledge could be useful to identify rare coding variants and genetic propensity eliciting SLE pathogenesis in Thais.

Note that some of the variants were previously characterized in other autoimmune diseases, including rheumatoid arthritis and primary Sjögren syndrome (pSS). It, therefore, indicates the sharing of underlying genetic factors between autoimmune disease. However, predisposing factors which could affect clinical manifestation driving different autoimmune disease outcome has not been elucidated yet. Recently, the GRS (genetic risk score) has been widely adopted to predict disease outcomes from genetic variants [68]. The previous studies in SLE showed that overall mortality was higher in the striking GRS SLE patients; also, the high cumulative genetic risk could predict the specific organ damages such as proliferative nephritis and cardiovascular disease [69]. Our study showed a high sensitivity for using polygenic risk scored as a marker for SLE disease development in the Thai population. It is exciting for further study to calculate the genetic risk score and specific clinical manifestation among Thai SLE patients.

### Conclusions

In conclusion, our study reported susceptible loci of SLE patients in Thai ancestry, which were variants on the HLA class II allele, *STAT4*, *GTF2I*, and *BLK*. Additionally, we confirmed those variants which had been reported previously in the Thai populations, which were *ARID5B*, *TNFSF4*, *BANK1*, *TNFAIP3*, *CXCR5 SLC15A*, *ITGAM*, *WDFY4*, and *ETS1*. Interestingly, we identified novel variants associated with the Thai SLE patients, which were on the *FNB2* allele*.* Summary loci associated with the Thai SLE were seen in Fig. 4b. Functional annotation analysis highlighted extracellular matrix organization pathways specific to the Thai population. The PRS using GWAS data is useful for SLE prediction with sensitivity and specificity of more than 70%. Further whole-genome sequencing study with a large sample size might help to validate our results. Finally, our finding provides the necessary genetic background susceptible to SLE disease, expanding the number of molecular targets for treatment options.

## Supplementary information

**Additional file 1.** Supplementary figure1: The graphs show quantile-quantile plot (Q-Q plot) with inflation factor value in observatory dataset (upper), and replication dataset (lower).

**Additional file 2.** Supplementary Table 1: List of significant variants at individual locus from meta-analysis (p-value < 5E-5)

**Additional file 3.** Supplementary Table 2: List of HWE of significant variants (p-value < 5E-5)

**Additional file 4.** Supplementary figure 2: The regional plots show variants found on *ATP6V1B1* (A), *ADCY5* (B), *DGKG* (C), *MIR4472-2* (D), and *MYO5C* allele (E). These SNPs were associated with Thai SLE patients with statistical significant value less than 10E-5.

## Data Availability

The complete results from the two Thai GWAS datasets and the GWAS meta-analysis are publicly available for download at http://2anp.2.vu/GWAS_SLE_Thailand.

## Abbreviations

GWAS: Genome-wide association study
SLE: Systemic lupus erythematosus
SNP: Single nucleotide polymorphism
HLA: Human leukocyte antigen
MHC: Major histocompatibility
CHR: Chromosome
MAF: Minor allele frequency
OR: Odds ratio
CI: Confidence interval
bp: Base pair
BCR: B cell receptor
TCR: T cell receptor
IL: Interleukin
PD: Program cell death
MDA: Melanoma differentiation-associated gene 5
